# Transcriptome Analysis and Single-Cell Sequencing Analysis Constructed the Ubiquitination-Related Signature in Glioma and Identified USP4 as a Novel Biomarker

**DOI:** 10.3389/fimmu.2022.915709

**Published:** 2022-06-14

**Authors:** Qikai Tang, Zhengxin Chen, Jiaheng Xie, Chuangqi Mo, Jiacheng Lu, Qixiang Zhang, Zhangjie Wang, Wei Wu, Huibo Wang

**Affiliations:** ^1^ Department of Neurosurgery, First Affiliated Hospital of Nanjing Medical University, Nanjing, China; ^2^ Jiangsu Key Lab of Cancer Biomarkers, Prevention and Treatment, Jiangsu Collaborative Innovation Center For Cancer Personalized Medicine, Nanjing Medical University, Nanjing, China; ^3^ Department of Burn and Plastic Surgery, The First Affiliated Hospital of Nanjing Medical University, Jiangsu Province Hospital, Nanjing, China; ^4^ Department of Neurosurgery, Pukou Branch of Jiangsu People’s Hospital, Nanjing Pukou District Central Hospital, Nanjing, China

**Keywords:** glioma, ubiquitination, bioinformatics, signature, immunotherapy

## Abstract

**Background:**

Glioma, the most frequent malignant tumor of the neurological system, has a poor prognosis and treatment problems. Glioma’s tumor microenvironment is also little known.

**Methods:**

We downloaded glioma data from the TCGA database. The patients in the TCGA database were split into two groups, one for training and the other for validation. The ubiquitination genes were then evaluated in glioma using COX and Lasso regression to create a ubiquitination-related signature. We assessed the signature’s predictive usefulness and role in the immune microenvironment after it was generated. Finally, *in vitro* experiment were utilized to check the expression and function of the signature’s key gene, USP4.

**Results:**

This signature can be used to categorize glioma patients. Glioma patients can be separated into high-risk and low-risk groups in both the training and validation cohorts, with the high-risk group having a significantly worse prognosis (P<0.05). Following further investigation of the immune microenvironment, it was discovered that this risk grouping could serve as a guide for glioma immunotherapy. The activity, invasion and migration capacity, and colony formation ability of U87-MG and LN229 cell lines were drastically reduced after the important gene USP4 in signature was knocked down in cell tests. Overexpression of USP4 in the A172 cell line, on the other hand, greatly improved clonogenesis, activity, invasion and migration.

**Conclusions:**

Our research established a foundation for understanding the role of ubiquitination genes in gliomas and identified USP4 as a possible glioma biomarker.

## Introduction

Glioma is the most frequent primary malignant tumor of the nervous system, accounting for 80% of all malignant tumors in the central nervous system and having a very bad prognosis (1). Gliomas are classified into four categories by the World Health Organization (WHO), with the first two types being low-grade gliomas (LGG) and the last two being high-grade gliomas (HGG) (2–4). Current conventional treatment options such as surgery, chemotherapy (temozolomide, etc.), and radiotherapy are still very limited in glioma (5). It is worth mentioning that the presence of the blood-brain barrier(BBB) has long been considered a challenge for the drug treatment of gliomas, to the extent that the FDA has only approved a few medications for the treatment of gliomas (6–8).. Glioma is also regarded as an immunosuppressive tumor, with the tumor microenvironment expressing and secreting a large number of immunosuppressive factors such as programmed cell death ligand-1 (PD-L1), cytotoxic T lymphocyte-associated protein 4 (CTLA-4), and Indolamine 2,3-dioxygenase (IDO), among others (9–11). The question of how to stimulate antitumor immunity in glioma is still being researched. Exploring the tumor microenvironment of glioblastoma and developing new biomarkers to aid prognostic assessment and therapy of glioma is so critical.

PTM (post-translational modification) is a covalent process in which proteins are sometimes modified by the addition of modifying groups and other times hydrolyzed to remove modifying groups, affecting their properties (12). The main forms of PTM include phosphorylation, glycosylation, acetylation, ubiquitination, carboxylation, ribosylation, and the pairing of disulfide bonds (13–15). Among them, ubiquitination is a widespread PTM mode that is considered to be highly correlated with autophagy (16, 17). E1 ubiquitin-activating enzyme activates the c-terminal glycine residue of ubiquitin-protein in an ATP-dependent way during ubiquitin modification, followed by E2 ubiquitin-conjugating enzyme and E3 ubiquitin ligase covalently attaching to the lysine (Lys) residue of the substrate protein (18–20). These substrates labeled by ubiquitin molecules are then recognized by the autophagy system and proteasome-mediated autophagy further occurs (21). Ubiquitination is a protein modification process widely existing in organisms, which is involved in homeostasis regulation and a series of pathophysiological processes (22). Ubiquitination, in particular, is expected to play a crucial role in cancer, as it regulates a variety of pathways and alterations in the microenvironment (23). Moreover, several key proteins involved in ubiquitination have been identified as promising targets for cancer therapy. Hence, it is time to explore the role of ubiquitination in glioma.

Now, bioinformatics analysis has provided us with new insights into cancer transcriptome changes (24). Through bioinformatics analysis, we can carry out cancer survival analysis and immune microenvironment analysis, thus providing new biological markers for the precise treatment of cancer. The most widely used databases are the TCGA and GEO databases, which are widely utilized for cancer bioinformatics analysis. In this study, we downloaded glioma data from TCGA database and GSE162631 data set from GEO database. Among them, GSE162631 is a single cell sequencing data set of glioma published in 2021, consisting of 4 tumor samples and 4 normal controls adjacent to tumors (25). In that study, Xie et al. revealed different states of brain endothelial cell (EC) activation and blood-brain barrier (BBB) impairment in gliomas.

In this study, we investigated the involvement of ubiquitination-related genes in glioma using bioinformatics analysis of glioma data. The ubiquitination-related prognostic signature was developed to separate glioma patients into groups, with the high-risk group having a much worse prognosis. Furthermore, in glioma, the ubiquitin signature can be used to identify changes in immune infiltration and immunological checkpoints. Our research will aid in the evaluation of glioma prognosis and treatment development.

## Methods

### Datasets Downloading and Filtering

We obtained RNA-seq data from the Cancer Genome Atlas (TCGA) database (https://portal.gdc.cancer.gov/) for glioblastoma (GBM) and lower-grade glioma (LGG). The following criteria were used to choose participants: (1) Patients with a past pathological diagnosis of lower-grade glioma or glioblastoma; (2) Gene expression and clinical data are reported for each patient. A total of 692 patients were included in the analysis after screening. Half of the patients were randomly assigned to the training cohort, while the other half was assigned to the validation cohort.

### Identification of Genes Associated With Ubiquitination

The GENECARDS database (https://www.genecards.org/) was used to find genes relevant to ubiquitination. All ubiquitination-related genes were found by searching for “ubiquitination” in the search box. For further investigation, we extracted the top 100 most relevant genes.

### Identification of Prognostic Ubiquitination-Related Genes

Univariate COX regression was used to identify genes linked with patient survival in gliomas in order to investigate the prognostic significance of these ubiquitin-related genes. The analysis platform is R software (version 4.1.0), and the “Survival” R package is utilized for COX regression analysis.

### Construction of the Prognostic Model

To build a prognosis model of ubiquitination, researchers used Least Absolute Selection and Shrinkage Operator (LASSO) regression after identifying ubiquitination genes having prognostic value. Each model gene was matched to generate the relevant coefficient after achieving the optimal LAMDA value, allowing the risk score of various patients to be calculated: Risk score =∑_(I =1)^nmβ _I *(expression of ubiquitination associated gene I). Based on the median risk value as a cut-off, patients in various cohorts might be separated into high-risk and low-risk categories. The model’s prognostic usefulness was then investigated using survival analysis to measure the prognostic difference between the two groups. The prognostic model’s 1, 3, and 5-year ROC curves were also generated to assess the model’s accuracy and robustness.

### Clinical Prediction Value of the Established Prognostic Model

To avoid bias, univariate and multivariate COX regression studies were done to further examine the model’s prognostic efficacy. To discover independent prognostic markers, risk scores and other clinical characteristics (age, sex, and Karnofsky performance score) were included in the analysis.

### Single-Cell Analysis of the Immune-Related Cellular Location of the Prognostic Associated Genes

To analyze the single-cell data acquired from the GEO databases, we utilized the “Seurat” software (version 1.3.1). The PCA dimension reduction approach, as well as the t-Distributed Stochastic Neighbor Embedding (tSNE) method, were used to identify cell subclusters in dataset GSE162631. Using feature genes and the “SingleR” packages, the cells were re-clustered. As a result, the expression of several cells was demonstrated.

### Immune Microenvironment Analysis

The tumor microenvironment and GBM/LGG-infiltrating immune cells were then assessed in silico. Based on bulk RNA-seq data, ESTIMATE is an algorithm for predicting the presence of invading stromal/immune cells in the tumor microenvironment. ESTIMATE was able to generate three scores based on single-sample Gene Set Enrichment Analysis (ssGSEA): stromal cell scores, immune cell scores, and ESTIMATE scores. CIBERSORT is a deconvolution technique that quantifies the proportions of distinct cell types by predicting the cellular composition of complicated tissues based on gene expression data. The link between risk score and tumor immune infiltration score was investigated using a total of seven methods.

### In Silico Prediction of Potential Antitumor/Cytotoxic Drugs

The R package “pRRophetic” is used to predict clinical chemotherapeutic response using tumor gene expression levels. pRRophetic is capable of forecasting possibly sensitive medications that are ideal for patients based on data obtained from a vast amount of data regarding the response of various tumor cell lines to anticancer drugs. We looked for medications that may be more effective in treating high-risk patients by utilizing pRRophetic to predict the IC50 of certain anticancer agents.

### Construcion of the Nomogram

Based on the results of multivariate cox regression, nomogram is a versatile approach of merging several risk factors into a single plot. We were able to visually forecast a patient’s survival probability using the nomogram created with the R packages ‘DynNom’.

### Cell Culture and Antibodies

American Type Culture Collection(ATCC) provided U87-MG and LN229 cells. Shanghai Institutes for Biological Sciences provided U251 and A172 cells (Shanghai, China). In four glioma cell line culture and *in vitro* investigations, Dulbecco’s Modified Eagle Medium (DMEM, gibco, CA, USA) with 10% fetal bovine serum (FBS) and 1% penicillin-streptomycin solution was utilized. Lonza provided normal human astrocytes (NHAs), which were cultivated in astrocyte growth medium containing rhEGF, insulin, ascorbic acid, GA-1000, L-glutamine, and 5% FBS. All of the cells were grown at 37°C with 5% CO2. Abcam provided antibodies against USP4, E-cadherin, and N-cadherin. Cell Signaling Technology provided the β-actin.

### Transfection

Ribobio (Guangzhou, China) developed and synthesized two distinct small interfering (si)RNAs against USP4. The target sequences of siRNA for USP4 were 5′- ACTGCAAAGTCGAGGTGTA-3′ (siUSP4-1) and 5′- GCAACACCTACGAGCAGTT-3′. (siUSP4-2). Genomeditech(Shanghai,China) designed and produced the USP4 over-expression plasmid. All transfections were carried out according to the manufacturer’s instructions using Lipofectamine 3000 (Invitrogen; Thermo Fisher Scientific, Inc.).

### Quantitative Real−Time Polymerase Chain Reaction (qRT-PCR)

Total RNA was extracted from cell lines with TRIzol reagent (Invitrogen, CA, USA) according to the manufacturer’s protocol. cDNA was synthesized with the PrimeScript RT Reagent Kit (Takara, Nanjing, China).qRT-PCR was implemented utilizing AceQ Universal SYBR qPCR Master Mix (Vazyme, Nanjing, China) on an ABI Stepone plus PCR system (Applied Biosystems, FosterCity, CA, USA). Primers used in this study were listed as follows: USP4(Forward): GCAGACACCATTGCAACCATC; USP4(Reverse): AACTGCTCGTAGGTGTTGCT. β-actin(Forward):GTCATTCCAAATATGAGATGCGT; β-actin(Reverse): GCATTACATAATTTACACGAAAGCA. Relative quantification was determined using the 2^-ΔΔCt^ method.

### Western Blotting

RIPA buffer with protease inhibitors (Roche) was used to lyse cellular proteins, and an equal amount of proteins was electrotransferred onto a polyvinylidene difluoride membrane (Millipore). The main and secondary antibodies were used to incubate the protein, which was then identified using enhanced chemiluminescence methods.

### CCK-8 Assay

Cell counting kit-8 test (CCK-8) was used to assess the proliferation capabilities of GBM cells (87-MG, LN229, and A172) according to the manufacturer’s instructions. 96-well plates were used to seed the transfected cells. 10 μl of CCK-8 reagent was added to the test well at 24, 48, 72, and 96 hours after transfection and incubated for 2 hours at 37°C away from light. At a wavelength of 450 nm, the absorbance was measured.

### Colony Formation Analysis

U87-MG, LN229, and A172 cells were transfected and maintained in 6-well plates for about 12 days. The cells were then stained with 0.1 percent crystal violet for 30 minutes before being rinsed with PBS. If the colonies were larger than 1 mm in diameter, they were counted.

### Migration and Invasion Assays

Cell migration and invasion were measured using transwell assays. In the upper chamber, 2×10^4^cells were cultivated in 200 μL media without serum, while in the lower chamber, 600 μL complete medium was supplied for the migration assay. According to the manufacturer’s protocols, additional Matrigel was employed for the invasion experiments (BD Biosciences, Bedford, MA, USA). Cells were fixed with 4 percent PFA and stained with 0.1 percent crystal violet solution after 24 hours of incubation at 37°C with 5% CO2.

### Wound Healing

Cells were grown in 6-well plates for 24 hours before being scratched with a sterile pipette tip (20 μL). Each wound was examined by inversion microscopy(Olympus, Japan) at 0 and 24 hours after rinsing the cells with PBS to remove cellular debris. To examine the cell migration capacity, the total wound area was analyzed using ImageJ software.

## Results

**Figure 1:**
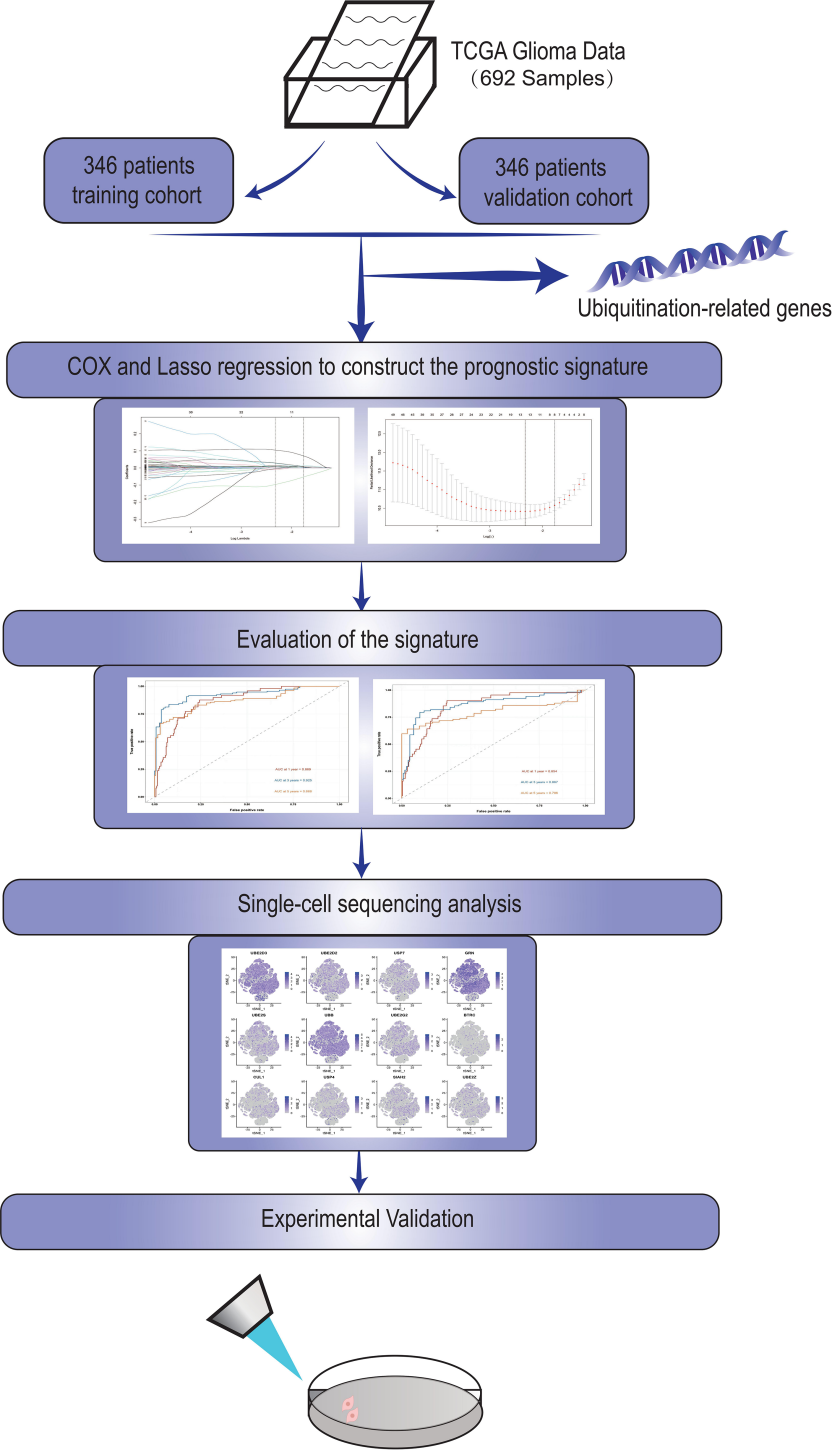
The work flow of our study.

Our flow chart is shown in **Figure 1**.

### Lasso Regression Was Performed to Construct a Ubiquitination-Related Signature in Glioma

**Figure 2:**
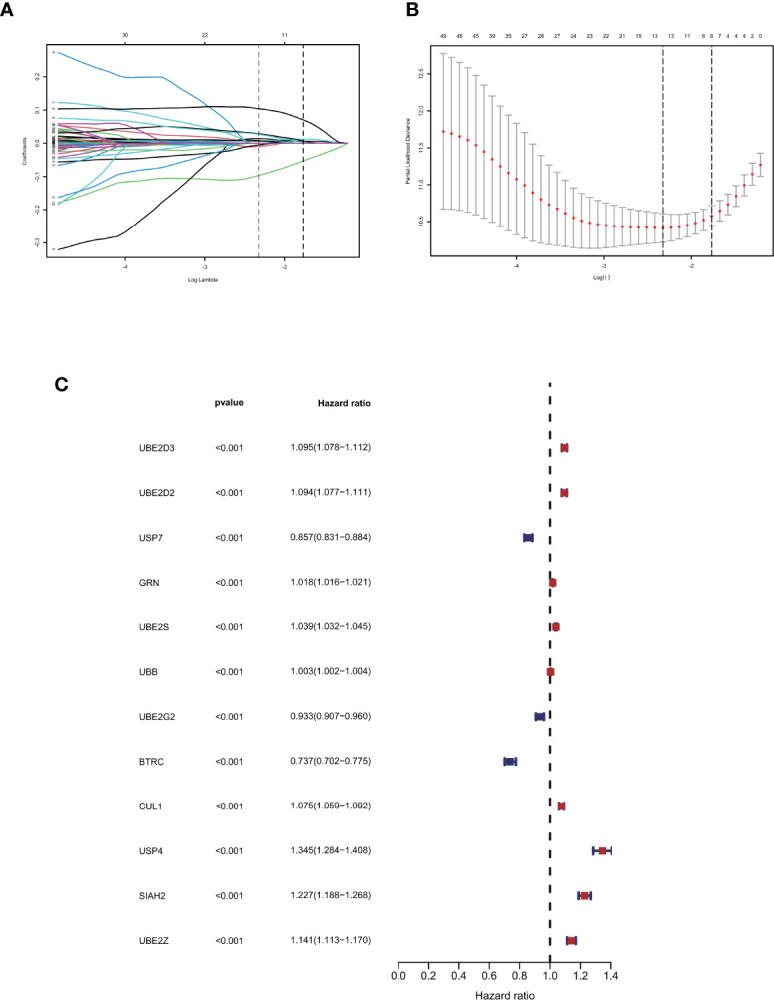
Identification of prognostic ubiquitination-related genes. **(A, B)** Lasso regression to construct the prognostic model. **(C)** COX regression of the 12 model genes.

**Figure 3:**
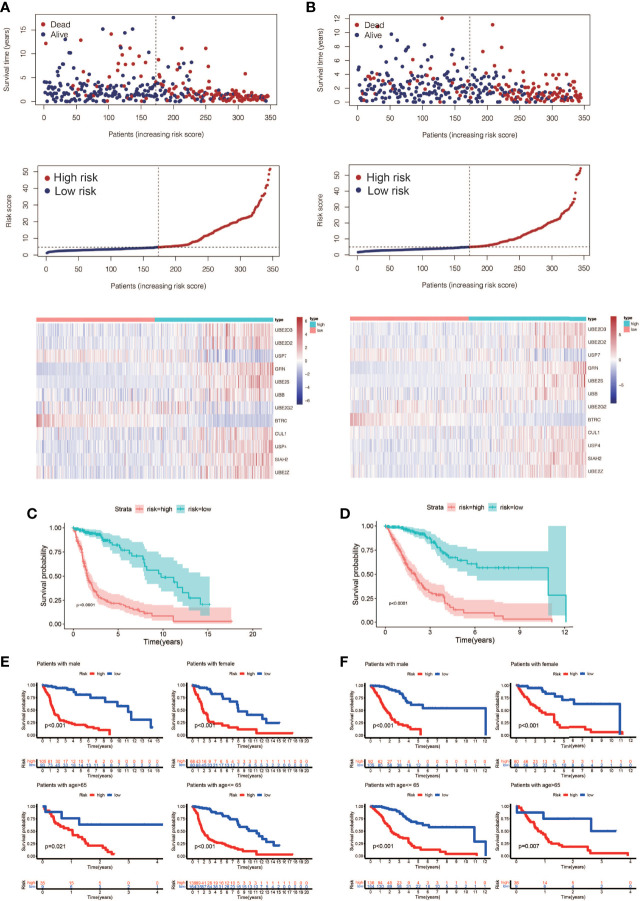
Prognostic model construction and evaluation. **(A)** The training cohort’s survival status, risk score, and model gene expression. **(B)** The validation cohort’s survival status, risk score, and model gene expression. **(C)** Analysis of the training cohort’s survival. The high-risk patients had considerably worse outcomes than the low-risk patients (P < 0.0001). **(D)** Analysis of the validation cohort’s survival. The high-risk patients had considerably worse outcomes than the low-risk patients (P < 0.0001). **(E)** A training cohort subgroup survival analysis. In different genders and age groups, a high risk score is linked to a poor prognosis. **(F)** Analysis of subgroup survival in the validation cohort. In different genders and age groups, a high risk score is linked to a poor prognosis.

A total of 72 ubiquitin-related genes with prognostic value in glioma were identified by univariate Cox regression of 100 ubiquitin-related genes obtained from Genecards database. Through Lasso regression of the above 72 genes, we obtained a Risk Score formula consisting of 12genes: Risk Score=0.0137868801576863*UBE2D3+0.00798080087559059*UBE2D2+(-0.00918452991096172)*USP7+0.0065644500970919*GRN+0.00751875352337762*UBE2S+0.000192089285965977*UBB+(-0.00497853632444545)*UBE2G2+(-0.0968360751270892)*BTRC+0.00754166909940448*CUL1+0.104123805908217*USP4+0.0289487761185436*SIAH2+0.0271749540402458*UBE2Z (**Figures 2A, B** **)**. Among them, UBE2D3, UBE2D2, GRN, UBE2S, UBB, CUL1, USP4, SIAH2, and UBE2Z were associated with poor prognosis of glioma(HR>1, P<0.001, **Figure 2C**). USP7, UBE2G2, and BTRC were associated with better prognosis of glioma(HR<1, P<0.001, **Figure 2C**). Using this formula, each patient can be calculated to obtain a risk score. Patients in different cohorts can be separated into high-risk and low-risk groups based on the median value. **Figure 3A** showed the survival status, score curve, and expression of model genes of the high-risk and low-risk groups of the training cohort. **Figure 3B** showsed the validation cohort analysis results. The dot plot of survival status, whether in the train cohort or the validation cohort, indicates that as the risk score grows, the survival time of patients gradually concentrates near the bottom, indicating a worse prognosis (**Figures 3A, B** **)**. Survival analysis on the training cohort showed that the high-risk group has a significantly worse prognosis than the low-risk group (**Figure 3C**). The same result was found in the survival analysis of the validation cohort (**Figure 3D**). Then the results of subgroup survival analysis on train cohort showed that high risk score is associated with poor prognosis in different genders and age groups (**Figure 3E**) and the results were verified in the validation cohort (**Figure 3F**).

### Univariate and Multivariate Cox Regression Were Used to Evaluate the Independent Prognostic Value of Risk Scores in Gliomas

**Figure 4:**
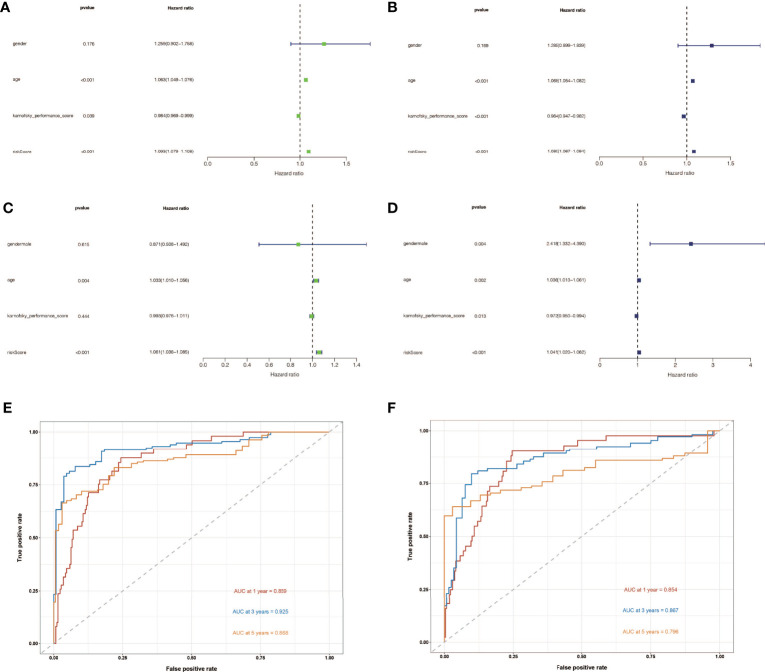
The independent prognostic significance of risk scores in gliomas was assessed using univariate and multivariate Cox regression. **(A)** In the training cohort, univariate Cox regression revealed that age and risk score are independent prognostic indicators of glioma. **(B)** In the validation cohort, univariate Cox regression revealed that age and risk score were independent prognostic indicators of glioma. **(C)** In the train cohort, multivariate Cox regression revealed that age and risk score were independent prognostic markers of glioma. **(D)** Multivariate Cox regression revealed that gender, age, and risk score were independent prognostic indicators of glioma in validation cohort study. **(E)** Training cohort’s ROC curve. The AUC for 1, 3, and 5 years was 0.869, 0.925, and 0.868, respectively. **(F)** ROC curve for validation cohort showed that the AUC of 1, 3 and 5 years were 0.854, 0.867 and 0.796, respectively.

To determine the independent prognostic usefulness of risk score, we used univariate and multivariate Cox regressions. First, univariate Cox regression in the training cohort revealed that age and risk score are independent prognostic predictors of glioma (**Figure 4A**). Age and risk score were also revealed to be independent prognostic predictors of glioma in validation cohort analysis using univariate Cox regression (**Figure 4B**). After that, the multivariate Cox regression was run. Age and risk score were determined to be independent prognostic predictors of glioma in the training cohort using multivariate Cox regression (**Figure 4C**). Gender, age, and risk score were confirmed to be independent prognostic predictors of glioma in a second validation cohort analysis using multivariate Cox regression (**Figure 4D**). We then created ROC curves for this signature in both the train and validation cohorts to assess its accuracy. The area under the curve (AUC) of 1, 3, and 5 years was 0.869, 0.925, and 0.868, respectively, according to the ROC curve of the training cohort (**Figure 4E**). The AUC of 1, 3, and 5 years was 0.854, 0.867, and 0.796, respectively, according to ROC curves for the validation cohort, demonstrating that the signature can accurately determine the prognosis of patients with glioma (**Figure 4F**).

### Immune Infiltration Analysis in High-Risk and Low-Risk Groups

**Figure 5:**
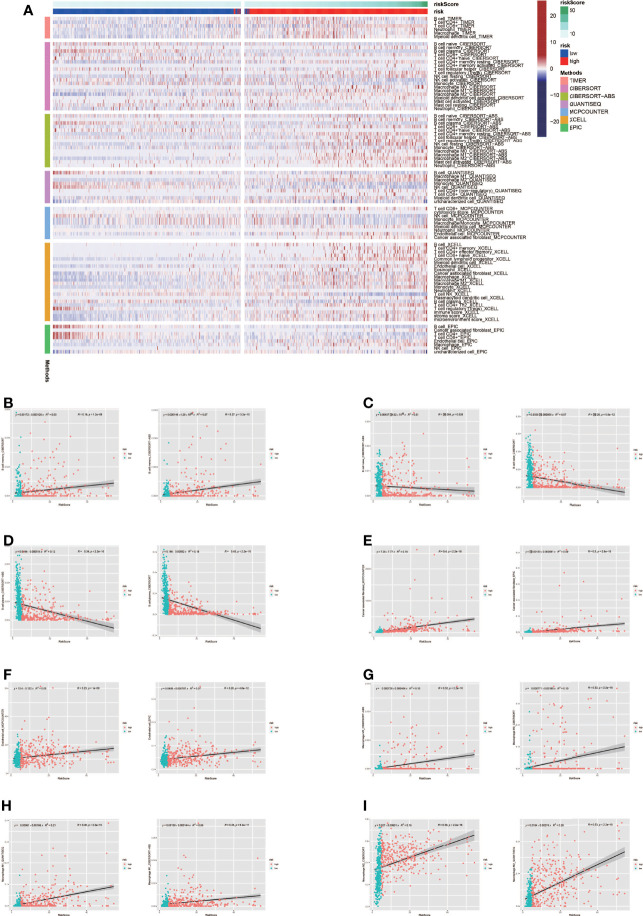
Analysis of immune microenvironment. **(A)** Immune landscape in high-risk and low-risk groups. The high-risk group tended to have higher levels of immune cell infiltration. **(B-I)** Many immune cells were significantly correlated with risk scores.

Tumor formation and progression are influenced by the immune microenvironment. Understanding the effects of the immunological microenvironment on tumor prognosis and treatment is beneficial. As a result, we looked at how immune infiltration differed between the high-risk and low-risk groups. To begin, we used multiple algorithms to create an immunological infiltraion heat map for high-risk and low-risk groups, with red representing high invasion levels and blue representing low invasion levels (**Figure 5A**). Following that, we conducted a correlation analysis between immune cells and risk ratings, finding that many immune cells were substantially connected with risk scores (**Figures 5B–I**).

### Analysis of Immune Checkpoint (ICP) and Microsatellite Instability (MSI)

**Figure 6:**
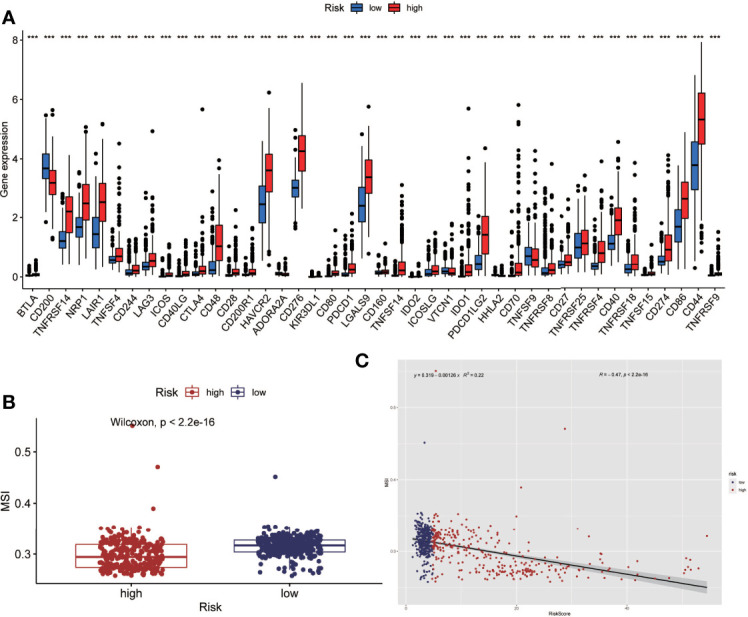
Analysis of immune checkpoint(ICP) and microsatellite instability(MSI). **(A)** The expression level of immune checkpoint related genes was higher in the high-risk group. **(B)** Microsatellite instability was lower in the high-risk group. **(C)** Correlation analysis also showed that microsatellite instability decreased as the risk score increased. (**P < 0.01, ***P < 0.001).

Tumor immunotherapy is a promising treatment option. Immune checkpoint-related gene expression and microsatellite instability are crucial indications for evaluating immunotherapy’s effectiveness. Between high-risk and low-risk groups, we looked at differences in immune checkpoint-related genes and microsatellite instability. The high-risk group had higher levels of expression of immune checkpoint genes, according to the findings (**Figure 6A**). The high-risk group had less microsatellite instability (**Figure 6B**). Microsatellite instability decreased as the risk score grew, according to correlation analyses (**Figure 6C**).

### Single-Cell Sequencing Analysis Based on Public Databases

**Figure 7:**
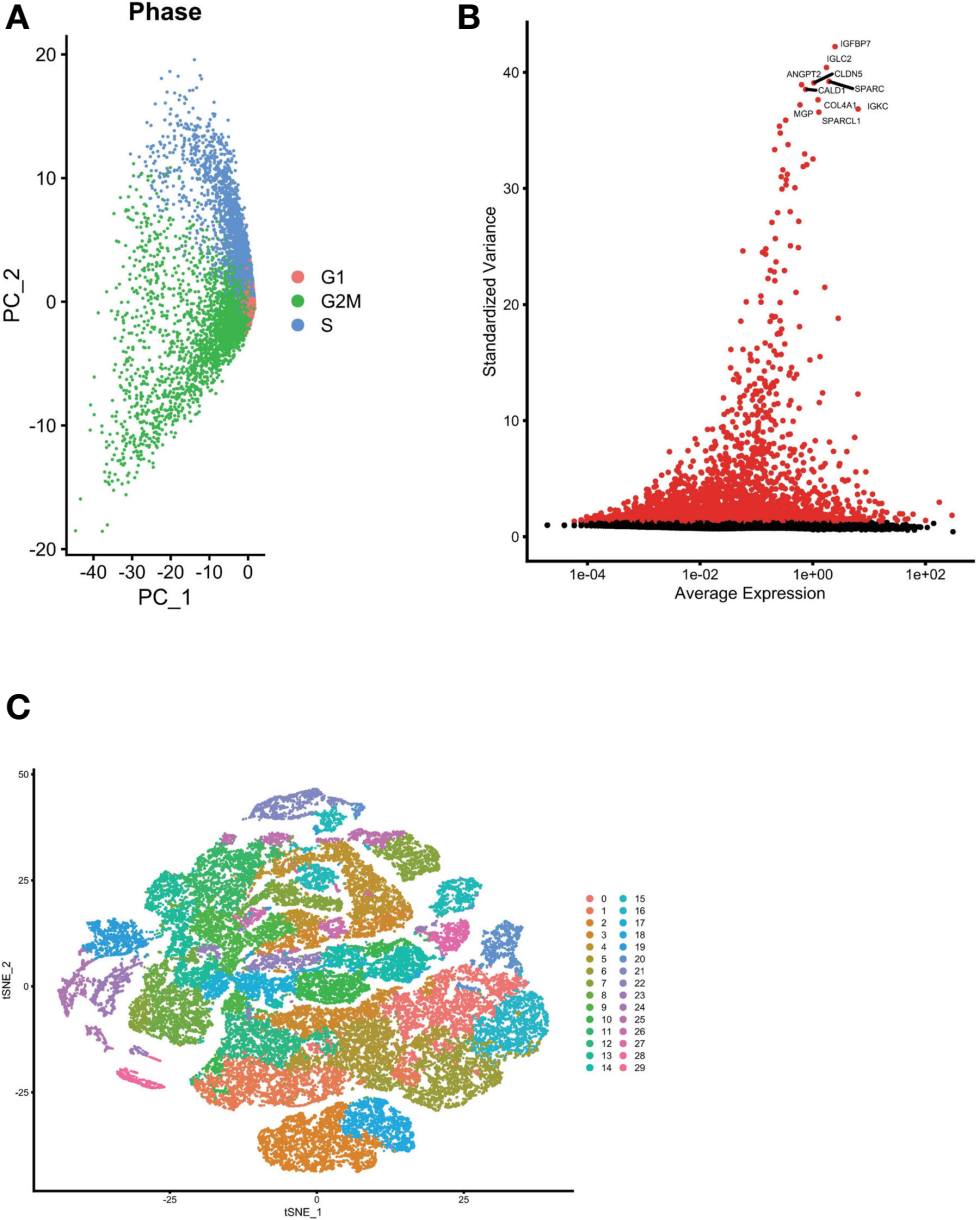
Single cell sequencing analysis was performed to reduce the dimensionality of glioma cells. **(A)** PCA reduction plot showed no significant differences in cell cycles. **(B)** The top 3000 variable features were labeled in red. **(C)** Dimensionality reduction and cluster analysis. The principle component analysis showed the distribution of the samples, and the results showed no significant batch effects.

**Figure 8:**
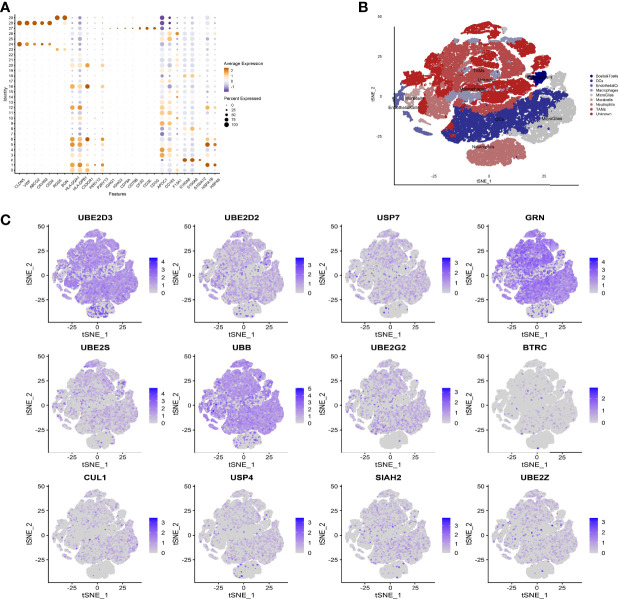
Expression of model genes at single cell level. **(A)** After dimension reduction and the tSNE clustering, the immune cells were identified using their feature genes. **(B)** The cells were divided into approximately 6 categories of cell types, namely endothelial cells(EC), neutrophils, T cells/B cells, mural cells, tumor-associated macrophages (TAMs), didenric cells and microglias. **(C)** The genes involved in the signature were identified in the single-cell tumor microenvironment atlas.

We used the Seurat package to process the single-cell transcriptome data. Raw data from GSE162631 were downloaded. A total of 4 GBM samples were downloaded for further analysis. The data with a mitochondrial RNA percentage larger than 0.10 were filtered, and we eventually acquired 51,449 cells that meet the standard. PCA reduction plot showed no significant differences in cell cycles (**Figure 7A**). Meanwhile, we selected the top 3000 variable features, which were labeled in red(**Figure 7B**). And the top 10 variable features were labeled. In **Figures 7C**, the principle component analysis showed the distribution of the samples, and the results showed no significant batch effects. After dimension reduction and the tSNE clustering, the immune cells were identified using their feature genes (**Figure 8A**). It could be clearly recognized that the cells were divided into approximately 6 categories of cell types, namely endothelial cells(EC), neutrophils, T cells/B cells, mural cells, tumor-associated macrophages(TAMs), didenric cells and microglias (**Figure 8B**). The genes involved in the signature were identified in the single-cell tumor microenvironment atlas(**Figure 8C** **).** In a fleeting glimpse, we could see the expression of the gene UBE2D3, UBE2D2, GRN, UBB was ubiquitous in immune-microenvironment in the GBM patients, and UBE2G2, CUL1, and USP4 showed moderate expression in immune cells. Besides, almost all ubiquitination associated genes were invariably expressed in TAMs and Dendritic Cells, indicating the strong correlation of ubiquitination within those immune cells.

### Drug Sensitivity Analysis

**Figure 9:**
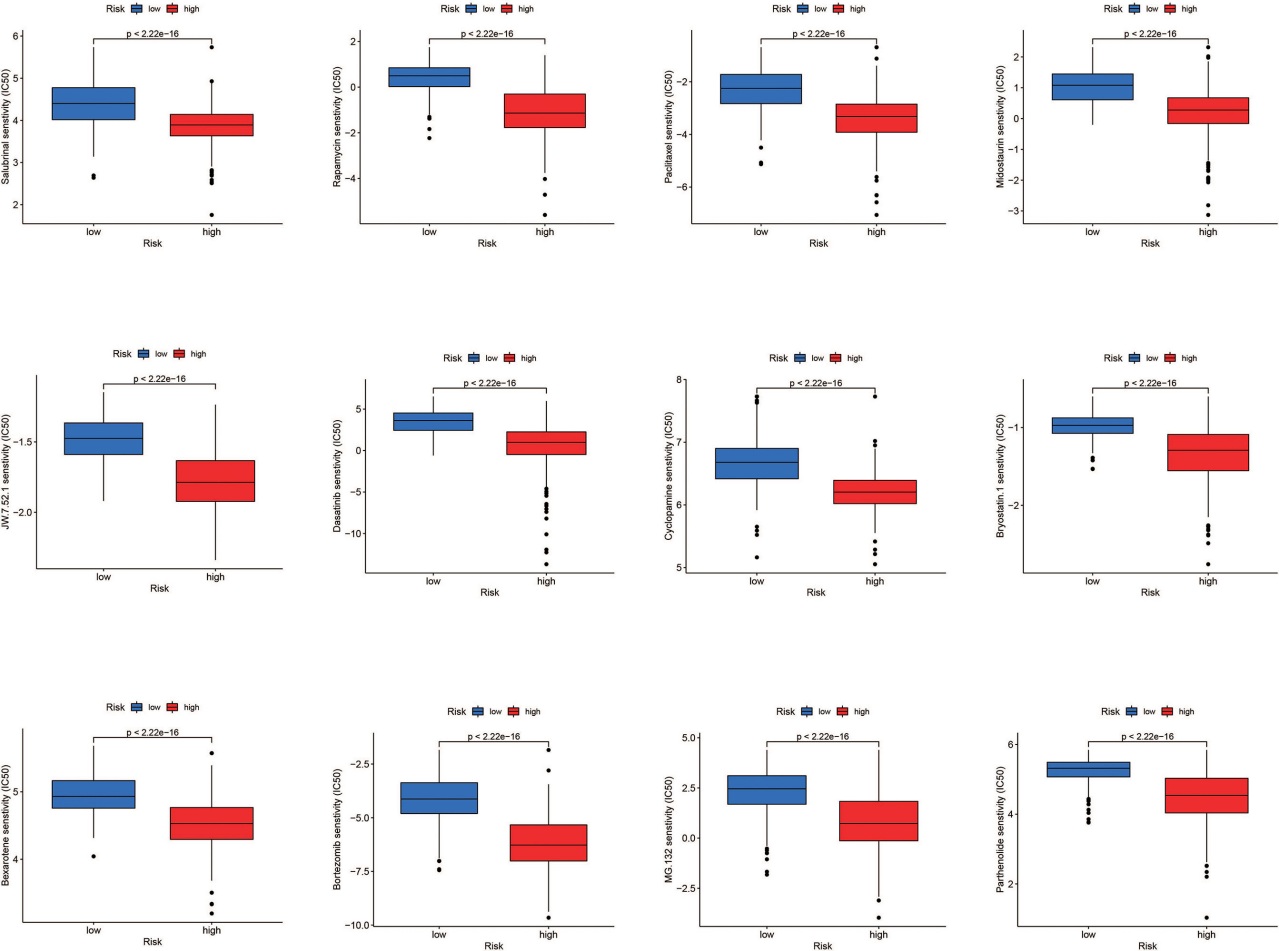
Drug sensitivity analysis. Salubrinal, rapamycin, paclitaxel, midostaurim, JW.7.52.1, dasatinib, cyclopamine, bryostatin.1, bexarotene, bortezomib, MG.132, and parthenolide had a lower IC50 in the high-risk group, meaning the high-risk group was more sensitive to these drugs.

According to the findings, high-risk patients have a worse prognosis. Therefore, in order to conduct precise intervention in high-risk patients, we performed drug sensitivity analysis to identify drugs that might be effective. Results showed Salubrinal, Rapamycin, Paclitaxel, Midostaurin, JW.7.52.1, Dasatinib, Cyclopamine, Bryostatin.1, Bexarotene, Bortezomib, MG.13 2., and Parthenolide had a lower semi-inhibitory concentration (IC50) in the high-risk group, meaning that the high-risk group was more sensitive to these drugs (**Figure 9**).

### The Nomogram Was Constructed to Further Evaluate the Prognosis of Glioma Patients

**Figure 10:**
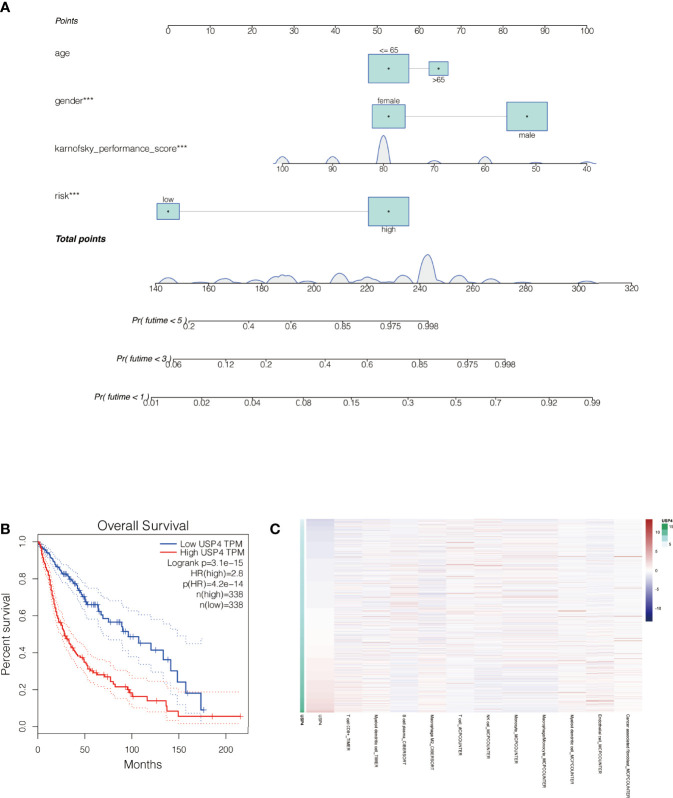
Nomogram of the signature and the analysis of the key gene USP4 in gliomas. **(A)** A nomogram was created to evaluate the prognosis of glioma patients at 1, 3, and 5 years. **(B)** Survival analysis of USP4 in GEPIA database. High expression of USP4 is associated with poor prognosis of gliomas. **(C)** Immune correlation analysis of USP4 in glioma. ***P<0.001.

By merging the clinical parameters of glioma patients, a nomogram was created to evaluate the prognosis of glioma patients at 1, 3, and 5 years **(** **Figure 10A** **)**.

### *In Vitro* Experiments Were Performed to Verify the Function of the Key Gene : USP4

**Figure 11:**
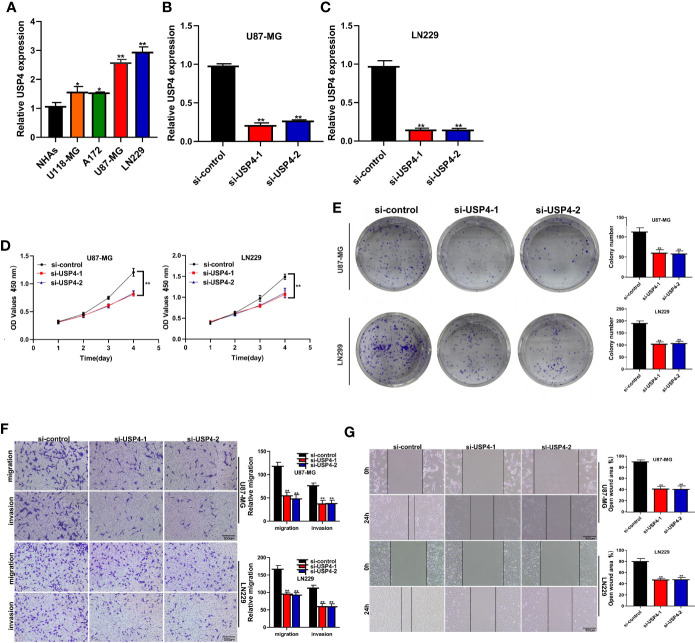
*In vitro* experiment after USP4 knockdown. **(A)** PCR test. Compared with NHAs normal control, USP4 was up-regulated in U118-MG, A172, U87-MG and LN229 glioma cell lines, with U87-MG and LN229 having the highest expression levels (*P < 0.05, **P < 0.01). **(B)** Then U87-MG and LN229 cell lines were transfected with siRNAs. **(C)** Both two siRNAs significantly down-regulated USP4 expression in U87-MG and LN229 cell lines (**P < 0.01). **(D)** CCK-8 experiments. After USP4 knockdown, the activity of U87-MG cell line and LN229 cell line decreased significantly. **(E)** After USP4 knockdown, the cloning ability of U87-MG cell line and LN229 cell line decreased significantly. **(F)** Transwell assay. After USP4 knockdown, the migration and invasion abilities of U87-MG cell line and LN229 cell line were significantly decreased. **(G)** Scratch test. After USP4 knockdown, the migration ability of U87-MG cell line and LN229 cell line decreased significantly.

**Figure 12:**
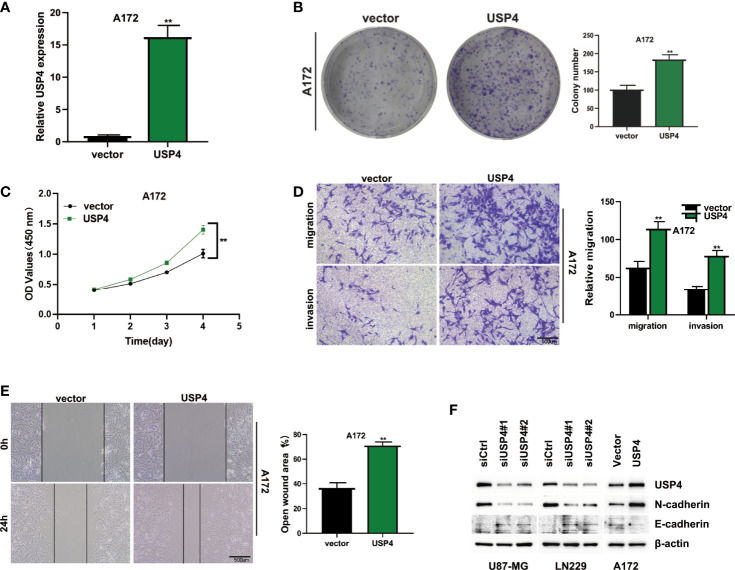
*In vitro* experiment after USP4 overexpression. **(A)** USP4 was overexpressed in A172 cell line after plasmid transfection (**P < 0.01). **(B)** Clonal formation experiments verified the changes of colony formation ability after USP4 overexpression (**P < 0.01). **(C)** CCK-8 assay showed that USP4 overexpression significantly enhanced the viability of A172 cell lines (**P < 0.01). **(D)** Transwell assay showed that USP4 overexpression significantly enhanced the ability of A172 cell line to migrate and invade (**P < 0.01). **(E)** Wound healing experiments showed that the migration ability of A172 cell line was significantly enhanced after USP4 overexpression (**P < 0.01). **(F)** Western-blotting assay was performed to verify the knockdown and overexpression efficiency of USP4 and explore the relationship between USP4 and EMT-related proteins.

The role of USP4 in glioblastoma was confirmed since its HR value was the highest in the signature. First, a survival analysis using the GEPIA database revealed that increased USP4 expression in glioma patients was linked to poor outcomes (**Figure 10B**). USP4 was found to be linked to a variety of immune cells in immunological investigation (**Figure 10C**). GSVA analysis of USP4 and ubiquitination related pathways is presented in **Supplemental Figure S1**. *In vitro* tests were then carried out to confirm USP4’s function. To begin, qRT-PCR revealed that USP4 expression was up-regulated in all four glioma cell lines when compared to normal control NHAs cell lines (**Figure 11A**), with the highest expression in U87-MG and LN229 cell lines, so gene knockdown was performed in these two cell lines (**Figure 11B**, *P<0.05, **P<0.01). In the U87-MG and LN229 cell lines, both siRNAs drastically reduced USP4 expression (**Figure 11C**). USP4 knockdown significantly reduced the activity of U87-MG and LN229 cell lines in the CCK-8 experiment (**Figure 11D**, **P<0.01). The ability of the U87-MG and LN229 cell lines to form colonies was dramatically reduced following USP4 knockdown (**Figure 11E**, **P<0.01). After knocking out USP4, the migratory and invasion capacities of the U87-MG and LN229 cell lines were dramatically reduced (**Figure 11F**, **P<0.01). The ability of U87-MG and LN229 cell lines to heal was dramatically reduced following USP4 knockdown (**Figure 11G**, **P<0.01). Following that, plasmid overexpressed USP4 in the A172 cell line (**Figure 12A**). The colony forming ability of the A172 cell line was greatly improved after overexpression of USP4 (**Figure 12B**, **P<0.01). The viability of the A172 cell line was dramatically improved following USP4 overexpression in the CCK-8 experiment (**Figure 12C**, **P<0.01). In a transwell experiment, USP4 overexpression greatly improved the A172 cell line’s migration and invasion capacity (**Figure 12D**, **P<0.01). In wound healing assays, USP4 overexpression greatly improved the migratory ability of the A172 cell line (**Figure 12E**, **P<0.01). The knockdown and overexpression efficiencies of USP4 were confirmed by Western-blotting, and the link between USP4 and EMT-related proteins N-cadherin and E-cadherin was investigated. A statistically significant association was established between USP4 and the EMT proteins E-cadherin and N-cadherin (**Figure 12F**). N-cadherin expression was dramatically reduced in the siUSP4-1 and SiUSP4-2 groups when the USP4 gene was knocked down in the U87-MG cell line, whereas E-cadherin expression was significantly raised. N-cadherin expression was greatly reduced when the USP4 gene was knocked down in the LN229 cell line, whereas E-cadherin expression was significantly increased. After USP4 was overexpressed in A172, N-cadherin expression was drastically increased, but E-cadherin expression was significantly decreased.

## Discussion

Glioma, the most frequent and difficult-to-treat malignant tumor of the central nervous system, has a significant impact on patients’ quality of life and places a significant cost on human health (26). Of these, glioblastoma and wild-type IDH are the most malignant subtypes and have a high mortality rate once diagnosed (27). Existing conventional treatments seem to have limited benefits in glioma (28). Postoperative recurrence and drug resistance are still a major problem in clinical management of glioma (29). The high heterogeneity and complex immune microenvironment of gliomas are considered to be the main reasons for poor prognosis and poor therapeutic effect (30). In the microenvironment of glioma, there exist crosstalk of multiple signaling pathways and biological mechanisms, leading to its continuous growth and development (31).

Ubiquitination, a frequent kind of post-translational protein modification, has been linked to cancer development (32). Ubiquitination alters intracellular protein interactions by degrading substrate proteins by proteasome (33). Since substrate proteins may be carcinogenic or suppressive, ubiquitination also plays a dual role in cancer (34). At present, many key enzymes in the ubiquitination process are considered as promising targets for cancer therapy (35). In addition, the importance of ubiquitination in glioma has been hypothesized. Chen et al. discovered that RNF139, an E3 ligase, plays a tumor suppressor role in glioma by modulating the PI3K/AKT signaling pathway and encouraging glioma cell apoptosis (36). Liang et al., on the other hand, discovered through cell studies that ubiquitin specific proteinase 22 (USP22) increased glioma cell proliferation, migration, and invasion, as well as promoting glioma growth and development (37). Thus, different members of the ubiquitination system may be foes or friends in gliomas. Detailed analysis of these members is needed to determine their role in glioma.

The role of ubiquitination-related genes in gliomas was investigated in this study. On the genecards database, 12 ubiquitin-related genes with prognostic significance were discovered using univariate Cox regression. Following that, Lasso regression of these 12 genes was used to create a predictive signature associated with ubiquitination in gliomas. A risk score for each patient can be determined using this signature. Based on the median risk value, glioma patients in the cohort can be split into two groups: high-risk and low-risk, with the high-risk group having a much worse prognosis than the low-risk group. This serves as a guide for glioma prognosis and risk assessment. Immune research revealed that the high-risk and low-risk groups had different amounts of immune infiltration. Furthermore, we can show that the high-risk group had a higher expression trend of immune checkpoint associated genes, but lower microsatellite instability. In addition, we mapped the expression of the genes in the signature in distinct cells using single-cell analysis. Finally, cell studies were utilized to confirm that USP4, the most important HR gene in the signature, was expressed and functioned in gliomas.

The GSE162631 dataset is made up of only one cell. The authors investigated the activation status of distinct brain epithelial cells (EC) in gliomas, as well as the status of blood-brain barrier disruption, in the original study of this data set. We used this data set to investigate the expression of 12 model genes at the single-cell level in our research. This serves as a guide to comprehending the function and heterogeneity of this prognostic model in various cells.

Although immunotherapy has achieved initial success in many solid tumors and is considered a landmark discovery in cancer treatment, its application in gliomas is still limited (38). Furthermore, our understanding of the glioma immune microenvironment is still insufficient. The presence of the blood-brain barrier(BBB) is thought to be a barrier to drug action in intracranial tumors, attenuating their efficacy (39). It should also be mentioned that gliomas have long been considered “cold” tumors with a high degree of immunosuppression (40). As a result, more research into the immune microenvironment of glioblastoma is required to offer a foundation for immunotherapy. Our research discovered that high-risk glioma patients exhibited a higher expression trend of immune checkpoint-related genes and less microsatellite instability. This serves as a reference for glioma immune stratification and can assist guide glioma immunotherapy.

Ubiquitin-specific Protease 4(USP4) is the gene with the highest HR in our constructed signature and is associated with poor prognosis in gliomas. Our cell tests revealed that USP4 was highly expressed in glioma, and that knocking down USP4 expression dramatically reduced the activity, invasion, and migratory ability of glioma cells. This adds to the evidence that USP4 has a function in gliomas. USP4, a cysteine protease from the DUBs family, is involved in deubiquitination in cells. Many prior research have suggested that USP4 has a function in malignancies. PAK5-DNPEP-USP4 increases the growth and progression of breast cancer, according to Geng et al., and overexpression of USP4 is linked to a poor prognosis in breast cancer (41). USP4 expression was similarly linked to increased breast cancer invasiveness, according to Cao et al. (42). Yang et al. discovered that the USP4/SMAD4/CK2 axis increases esophageal cancer progression (43). USP4 was also discovered to be a potential target for gliomas in our research.

In conclusion, patients can be adequately classified and immunologically assessed using the ubiquitin-related prognostic signature in gliomas. Our research could lead to new approaches to glioma detection and therapy.

## Data Availability Statement

The original contributions presented in the study are included in the article/**Supplementary Material**. Further inquiries can be directed to the corresponding authors.

## Author Contributions

QT, JX, and HW contributed conception and design of the study; CM, JX, and QT collected the data; JX and QT performed the statistical analysis; QT, WW, and HW wrote the first draft of the manuscript. All authors contributed to manuscript and approved the submitted version.

## Funding

This work was supported by the National Natural Science Foundation of China (82072783, 81772681, 81670153, and 81872058), the Natural Science Foundation of Jiangsu Province (BK20160098), the Program for Development of Innovative Research Team in the First Affiliated Hospital of NJMU, and the Priority Academic Program Development of Jiangsu Higher Education Institutions.

## Conflict of Interest

The authors declare that the research was conducted in the absence of any commercial or financial relationships that could be construed as a potential conflict of interest.

## Publisher’s Note

All claims expressed in this article are solely those of the authors and do not necessarily represent those of their affiliated organizations, or those of the publisher, the editors and the reviewers. Any product that may be evaluated in this article, or claim that may be made by its manufacturer, is not guaranteed or endorsed by the publisher.
