# Supplementary figures and images for: Transcriptome Analysis and Single-Cell Sequencing Analysis Constructed the Ubiquitination-Related Signature in Glioma and Identified USP4 as a Novel Biomarker
Source: Front Immunol. 2022 Jun 14;13:915709. doi: 10.3389/fimmu.2022.915709 (PMC9238360; doi:10.3389/fimmu.2022.915709)

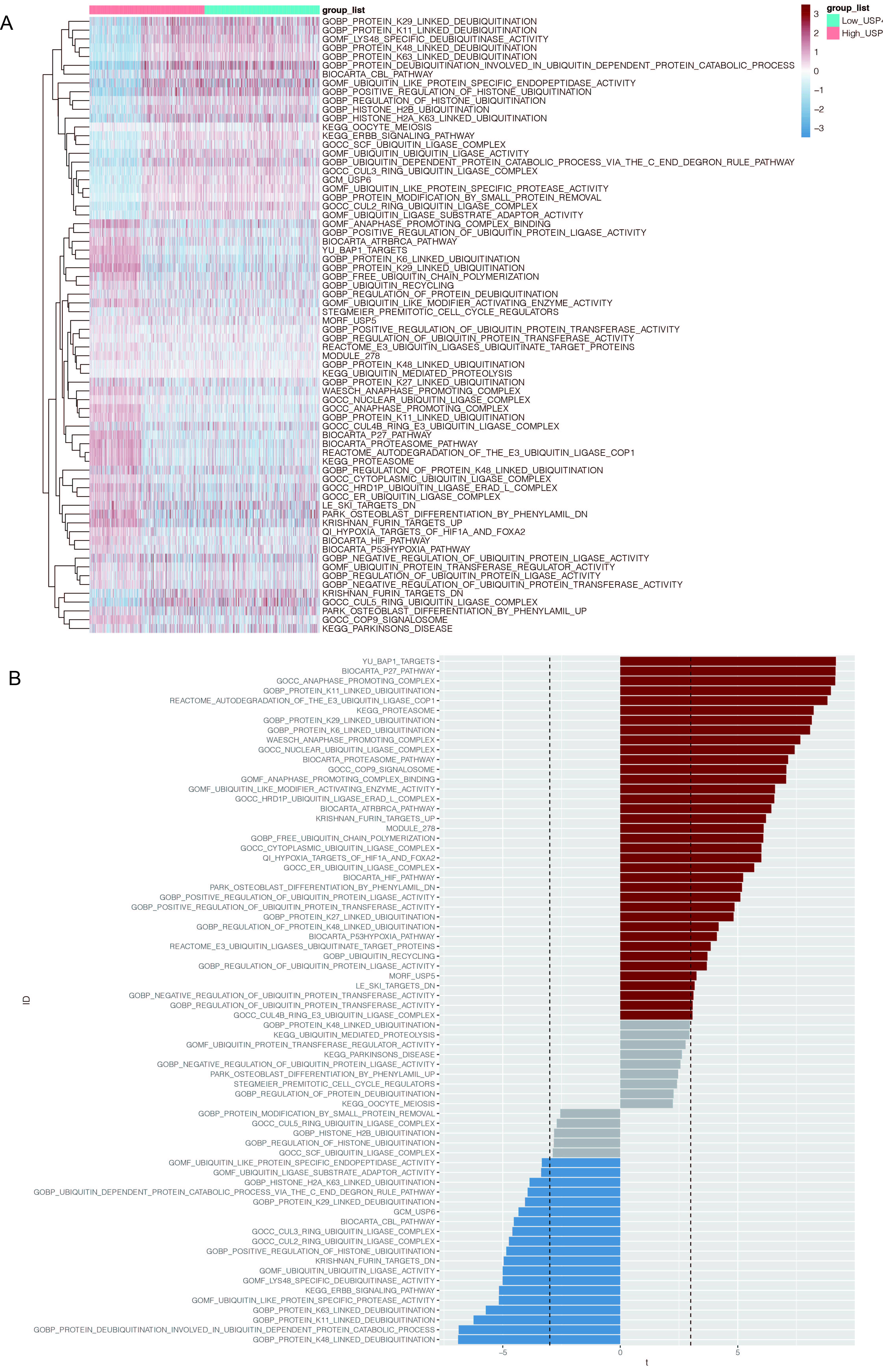

Supplement: Supplementary file 1 [file Image_1.jpeg]
